# Multidimensional Analysis of SARS-CoV-2 RNA in Nine Sites Located in Campania Region, Italy

**DOI:** 10.3390/microorganisms14051063

**Published:** 2026-05-08

**Authors:** Annalisa Lombardi, Patrizia Riccio, Maria Ragosta, Mariagrazia D’Emilio, Dario Bruzzese, Vito Imbrenda, Tonia Borriello, Giuseppina La Rosa, Elisabetta Suffredini, Ida Torre, Francesca Pennino

**Affiliations:** 1 Department of Public Health, University “Federico II”, Via Sergio Pansini 5, 80131 Naples, Italy; annalisa.lombardi@unina.it (A.L.); dbruzzes@unina.it (D.B.); tonia.borriello@unina.it (T.B.); ida.torre@unina.it (I.T.); 2 Department of Molecular Medicine and Medical Biotechnology, University “Federico II”, Via Sergio Pansini 5, 80131 Naples, Italy; patrizia.riccio@unina.it; 3 Department of Health Sciences, University of Basilicata, Viale dell’ Ateneo Lucano 10, 85100 Potenza, Italy; maria.ragosta@unibas.it; 4 Institute of Methodologies for Environmental Analysis, Italian National Research Council IMAA CNR, UDR Napoli, Corso Nicolangelo Protopisani, 80146 Naples, Italy; mariagrazia.demilio@cnr.it; 5 Institute of Methodologies for Environmental Analysis, Italian National Research Council IMAA CNR, Contrada Santa Loja, Tito, 85050 Potenza, Italy; vito.imbrenda@cnr.it; 6 National Center for Water Safety (CeNSiA), Istituto Superiore di Sanità, Viale Regina Elena 299, 00161 Rome, Italy; giuseppina.larosa@iss.it; 7 Department of Food Safety, Nutrition and Veterinary Public Health, Istituto Superiore di Sanità, Viale Regina Elena 299, 00161 Rome, Italy; elisabetta.suffredini@iss.it

**Keywords:** SARS-CoV-2, wastewater, epidemiology, multivariate analysis, cluster analysis

## Abstract

Wastewater monitoring has been recognized as a valid tool for monitoring coronavirus disease 2019 (COVID-19) diffusion. In this paper we analyse a dataset composed by the measurements of SARS-CoV-2 RNA load in 605 raw wastewater samples collected from nine wastewater treatment plants (WWTPs) in the Campania region from October 2021 to May 2025. We analyse the correlation structure of the dataset using multivariate statistical techniques with the aim of identifying the most representative sentinel WWTPs and thus optimizing the number of samples. Results of spatial analysis showed that there are two isolated elements, SA3 and NA1, with the highest and lowest SARS-CoV-2 load values, respectively, and other two clusters (Cl1 and Cl2) from the other WWTPs. Temporal analysis showed that NA3 WWTP had a statistically significant difference in SARS-CoV-2 load from 2022 to 2023. Our method suggests limiting samplings to three sites, as follows: SA3 (which can act as a sentinel site because it is the first site that records variation in viral load) and two with the higher variation coefficients (CV%) belonging to the two clusters, as follows: CE1 for Cl1 and NA4 for Cl2. This data analysis procedure could allow to focus only on certain WWTPs for SARS-CoV-2 monitoring, to promptly identify outbreaks.

## 1. Introduction

Wastewater-Based Epidemiology (WBE) is an approach based on the analysis and interpretation of biological and chemical markers in wastewater to infer population-level exposure, health status, and circulation of pathogens [1]. To explain the importance of WBE, the example of an iceberg representing infections can be used, as follows: clinical surveillance, which monitors hospitalisations, cases and deaths, identifies only the visible part of the iceberg, while environmental surveillance, which monitors the circulation of a pathogen even in the absence of clinical tests, identifies the visible and invisible part of that iceberg [2].

Examples of biomarkers that can be found in wastewater include pathogenic bacteria such as *Klebsiella pneumoniae*, *Pseudomonas aeruginosa*, and *Acinetobacter* spp., bacteriophages, antimicrobial resistance markers, and viruses [1].

Different infectious viruses, such as norovirus, enterovirus, adenovirus, rotavirus, astrovirus, monkeypox, and hepatitis A and E viruses, have been monitored through the WBE approach [3]. SARS-CoV-2 can also be monitored in wastewater, as it is present in the faeces of infected individuals and from there reaches wastewater treatment plants (WWTPs), where it undergoes a reduction in terms of numbers and viability [4].

From February 2020 to 5 November 2025, a total of 27.015.867 cases were registered in Italy. This number refers to the total number of people who have tested positive for SARS-CoV-2 since the start of the epidemic, comprising the sum of current cases, recovered cases, and deaths [5].

In 2021, a national wastewater surveillance programme for SARS-CoV-2 coordinated by the Italian National Institute of Health (Istituto Superiore di Sanità-NIH) was established, following the publication of a document endorsing the introduction of this surveillance by EU Member States [6]. In Italy, this project is known as SARI (Environmental Surveillance of Wastewater in Italy), and is focused on investigating SARS-CoV-2 and its variants in wastewater of regions and autonomous provinces that have decided to be part in the initiative.

Since wastewater is the result of the viral discharges by the population [1], a temporal correlation between SARS-CoV-2 load in wastewater and cases in the population exists, and it was investigated in recent years in different studies [7,8,9,10].

To obtain a detailed overview of the epidemiological situation over a large area such as an entire region, however, it is necessary to monitor numerous WWTPs serving different municipalities. This, however, requires the collection, transport, and analysis of numerous samples. Identifying WWTPs with specific characteristics that provide better information about SARS-CoV-2 circulation in the population than others could allow for a reduction in the number of samples collected and a focus predominantly on these WWTPs. For this reason, the selection of sites that reliably represent the epidemiological situation can be useful to provide information on infections trends in the population, while ensuring sustainability of the surveillance systems.

Thus, the aims of this study were the following: (a) to monitor SARS-CoV-2 presence in wastewater of nine WWTPs located in the Campania region, and (b) to perform a spatial and temporal analysis in order to assess whether a site can be used as a sentinel for the presence of SARS-CoV-2 in the future.

To our knowledge, this is the first study conducted in Campania (and, more generally, one of the few in Italy) that attempts to optimise the selection of the types of WWTP to be monitored for the presence of SARS-CoV-2.

## 2. Materials and Methods

### 2.1. Study Area and Wastewater Sampling

Six hundreds and five raw wastewater samples were collected by the Campania Regional Environmental Protection Agency (ARPAC) from nine WWTPs located in the Campania region: Napoli Est (NA1), Napoli Ovest—Main entrance (NA2), Napoli Ovest—North sewer (NA3), and Area Nolana (NA4) for the Naples area; Area Casertana (CE1) and Villa Literno (CE2) for the Caserta area; and Salerno (SA1), Nocera Sup. (SA2), and Eboli (SA3) for the Salerno area. Napoli Ovest—Main entrance and Napoli Ovest—North sewer belong to the same WWTP that has two different inlets that collect water from different municipalities.

**Figure 1:**
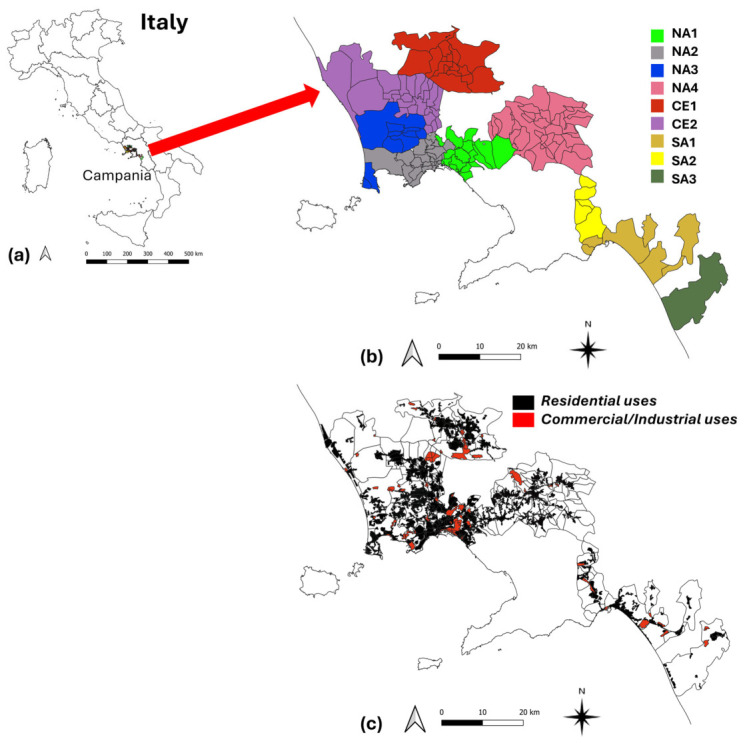
(**a**) Italy divided into its different regions (NUTS2 level), including Campania, which is the study area; (**b**) aggregation of municipalities or neighbourhoods (for the city of Naples only) served by the different WWTPs (for the understanding of the acronyms, see Table 2); (**c**) presence, within each area served by a specific WWTP, of residential areas and commercial/industrial areas.

To characterize each area served by the respective WWTP, the extents of residential and commercial/industrial surfaces were calculated using the CORINE Land Cover (CLC) 2018 map [11] at the third level of detail as the reference land use/cover dataset, available as shapefile. This map adopts a relatively detailed labelling of the urban component (see e.g., Ragosta et al. [12]), which includes continuous and discontinuous urban fabric (classes 111 and 112), considered in this study as residential urban fabric for the investigated areas (Table 1). Commercial and/or industrial areas consist of the so-called “Industrial or commercial units” (class 121), port areas (class 123), and airports (class 124). The other classes listed in the table were not included in either of the two macro-categories, as they generally do not generate discharges that can be collected by the hydraulic infrastructures conveying flows to the WWTP. The analysis of the CLC datasets was carried out through geospatial tools in GIS environment (QGIS 3.40.14) [13] (Figure 1).

The sampling was conducted from October 2021 to May 2025. In particular, samples from NA1, NA2, and NA3 were collected from October 2021 to May 2025; samples from NA4, CE1, and SA2 were collected from October 2021 to March 2023; samples from SA1 were collected from December 2021 to March 2023; and samples from SA3 were collected from February 2022 to March 2023. Samples were collected, with different sampling frequency. The variation in sampling frequency over time was related to the evolution of the surveillance programme and resource optimisation during the transition from the pandemic to the post-pandemic phase. Sampling frequency was once a week from October 2021 to December 2022, fortnightly in January and February 2023, and then once a month from March 2023 to May 2025. During the period November 2023–January 2024, samples were not collected. In Table 2 all available data are shown.

500 mL of the wastewater samples were collected in polyethylene bottles through automated samplers, to obtain 24 h composite samples that were transported to the laboratory at 4 °C within 2 h.

### 2.2. Samples Processing and Nucleic Acids Extraction

Samples were processed as described in La Rosa et al. [14]. Briefly, samples were disinfected externally and treated at 56 °C for 30 min to inactivate viral particles and protect personnel safety. The analysis was conducted on 45 mL of sample, to which 100 µL of Murine Norovirus provided by NIH (Rome, Italy) was added as a control for concentration and extraction efficiency. Concentration step was performed by centrifuging the samples at 4 °C, 4500× *g* for 30 min, by recovering 40 mL of supernatant and transferring it into tubes containing 4 g of polyethylene glycol (PEG, ThermoFisher, Kandel, Germany) and 0.9 g of sodium chloride (NaCl, Oxoid, Basingstoke, United Kingdom). Once the PEG and NaCl had dissolved in the supernatant, samples were centrifuged at 4 °C, 12.000× *g* for 2 h. Nucleic acid extraction was performed using the eGeneUP platform (bioMérieux, Marcy-l’Étoile, France), and elution was performed in 100 µL of TE elution buffer solution, pH 8.0. Nucleic acids were further purified by OneStep PCR Inhibitor Removal Kit (Zymo Research, Irvine, CA, USA), and RNA samples were stored at −80 (±10) °C or used instantaneously.

### 2.3. Real-Time RT-qPCR

SARS-CoV-2 analysis was done by detection of ORF-1b (nsp14, 3′-to-5′ exonuclease) through real-time RT-qPCR. The 25 µL mix consisted of AgPath-ID One-Step RT-PCR Reagents (Applied Biosystems, Waltham, MA, USA), primers and probes according to La Rosa et al., samples, and nuclease-free water [14]. Thermal steps were 50 °C for 30 min, 95 °C for 10 min, and 45 cycles of 95 °C for 15 s and 60 °C for 45 s. Each reaction was performed in technical duplicate. An RNA of SARS-CoV-2 provided by the NIH was used as a positive control, to assess PCR efficiency and as an external inhibition control. Results were expressed in genomic copies (g.c.)/µL of RNA by constructing a calibration curve with quantified dsDNA of SARS-CoV-2 provided by NIH. Results were converted to g.c./L of sewage and to g.c./day x inhabitant (SARS-CoV-2 load) considering the daily WWTP flow rate (that was explicitly accounted for in the analysis, thereby addressing dilution effects).

### 2.4. Data Analysis

In order to highlight different behaviours among the nine sampling sites, we selected the periods maximizing the data available for the spatial analysis. We considered a matrix, without data missing, of M = [9 sampling sites, 43 sampling days] from 17 February 2022 to 14 March 2023. For each sampling site, descriptive statistics were calculated. Our spatial analysis framework consists of three consecutive steps. As a first step, we perform a cluster analysis without applying preliminary tests. We use clustering as an explorative tool. The robustness and interpretability of the resulting clusters are subsequently supported by principal component analysis (PCA) and by an analysis of the corresponding centroids. On the correlation matrix (we calculated correlation coefficient for each pair of sites), an agglomerative hierarchical clustering (AHC) algorithm with complete linkage was applied. The homogeneous groups of sites (clusters) and any isolated elements are identified through the analysis of the dendrogram. For results validation, each cluster has to be characterized by means of endogenous indices (centroids are the mean values of descriptors measured in the cluster) [15]. Furthermore, it is possible to combine PCA data analysis applied to the same correlation matrix, determining the percentage of explained variance by different element or subgroups and their scores in space of principal components. @XLSTAT software (trial version) is used for multivariate analysis.

To link demographic and land use data with epidemiological information, we introduce an external variable, the *I_RES_* index, defined as

$$I_{RES}=\frac{A_{RES}}{N_{eqI}}$$

in which *A_RES_* is the residential area extension and *N_eqI_* is the number of equivalent inhabitants estimated for each sampling site. This index represents the effective residential surface available for equivalent inhabitant and is intended to capture residential crowding at the area level. In the methodological context, an external index may be included as a criterion for external validation; relationships among cluster and proxy indicator may provide support for the interpretation of the correlation structure.

Viral load during the three years of monitoring was summarized using descriptive statistics including median and interquartile range (IQR), along with the proportion of non-detectable samples.

Differences between years were assessed using generalized least squares (GLS) models to account for the longitudinal nature of the data according to the following model:

$$log\left(Y_{it}+1000\right)=\beta_{0}+\beta_{i}{\mathrm{Year}}_{i}+f\left({DOY}_{it}\right)+\varepsilon_{it}$$

where $Y_{it}$ denotes the viral concentration at time *t* for year *i*, and $f\left({DOY}_{it}\right)$ represents a smooth function of the day of the year modelled using natural cubic splines with 4 degrees of freedom. To account for temporal dependence, the residuals $\varepsilon_{it}$ were assumed to follow a continuous autoregressive correlation structure of order 1. In GLS models, SARS-CoV-2 loads were log-transformed after adding a constant (log(concentration + 1000)) to accommodate non-detectable values, but results were robust to the choice of this shift. Models were fitted using restricted maximum likelihood (REML). Diagnostic assessment was based on normalized residuals versus day of the year, Q–Q plot of normalized residuals, and autocorrelation function of normalized residuals.

Statistical significance was evaluated using two-sided tests, with *p*-values < 0.05 considered statistically significant.

## 3. Results

### 3.1. Spatial Analysis

**Figure 2:**
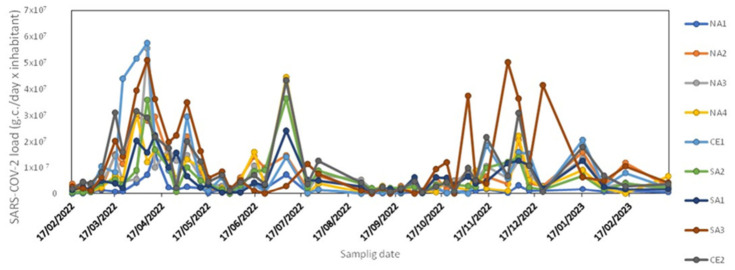
SARS-CoV-2 load in all monitored WWTPs.

Figure 2 shows the temporal trend of the data collected at the nine sampling sites from 17 February 2022 to 14 March 2023. The data show similar patterns, with peaks occurring at the same times across all sites, except for the values recorded from June 2022 onwards at the SA3 (higher than those of other WWTPs).

**Figure 3:**
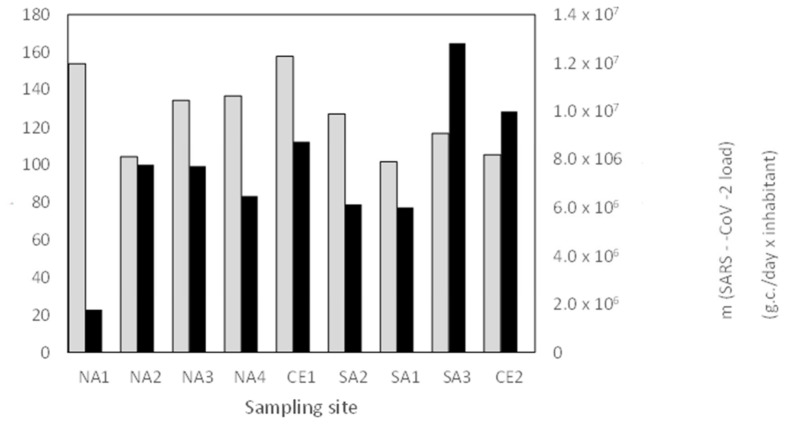
Means of SARS-CoV-2 load (black bars) and CV% (grey bars) for each sampling site.

We calculated the descriptive statistical parameters including mean values and coefficients of variation (CV%) in the same period. Figure 3 shows means and CV% for each site. The NA1 site has the lowest mean load value (0.2 × 10^7^ g.c./day x inhabitant) and a very high CV% (154%), whereas SA3 and CE2 have the highest mean load values (1.3 × 10^7^ g.c./day x inhabitant and 1.0 × 10^7^ g.c./day*inhabitant, respectively) and CV% of 117% and 105%, respectively. Interesting is the behaviour of CE1, which shows a CV% higher than 150%, similar to NA1, but has mean load values higher than other sites excluding SA3 and CE2.

**Figure 4:**
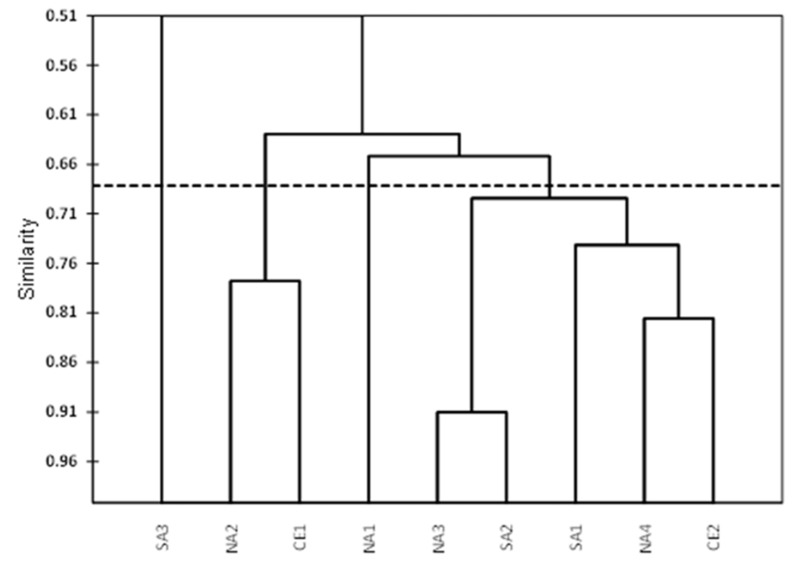
Dendrogram: classification based on SARS-CoV-2 load. The dashed line is the optimal cut line.

Figure 4 shows the dendrogram obtained by means of cluster analysis (the optimal line for the cut is shown). The cluster analysis highlights an isolated element, SA3, that remains isolated regardless of how the cut is made. Another isolated element, NA1, is put in evidence by the optimal cut at 0.67 of similarity. Moreover, this cut puts in evidence also two clusters Cl1—(NA2—CE1) and Cl2 (NA3, SA2, SA1, NA4, and CE2).

**Figure 5:**
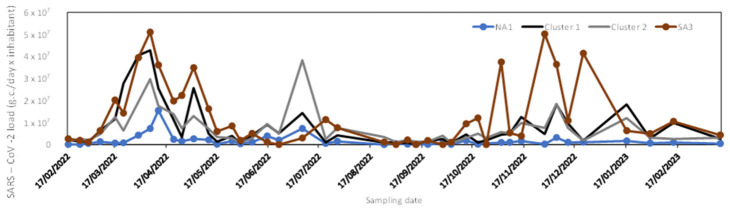
Mean values of SARS-CoV-2 load measured in the two isolated elements and in the two clusters.

The centroid patterns for the two clusters and the SARS-CoV-2 load values measured in the two isolated elements are shown in Figure 5. The separation between the two isolated elements (SA3 and NA1) compared to the cluster centroids is evident. Specifically, the two isolated elements represent the locations where the lowest values were measured (NA1) and where the highest values were recorded (SA3). The two clusters, Cl1—(NA2, CE1) and Cl2 (NA3, SA2, SA1, NA4, and CE2), occupy an intermediate position in virus load measurements. As shown in Table 3, the two isolated elements explain respectively: SA3 9.8% of the data variance (II PC) and NA1 6.6% (III PC), while all the others together in the I PC explain 67.8% of the variance.

**Figure 6:**
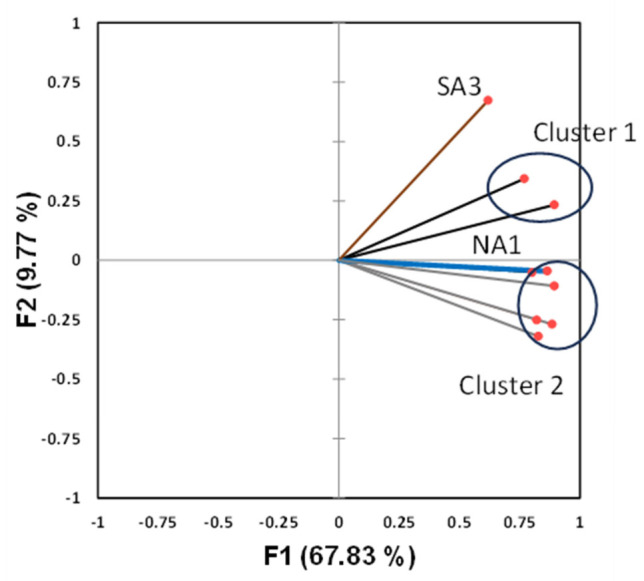
Results of PCA Analysis, in parentheses for each axis; the percentage of explained variance is shown.

Furthermore, the PCA score plot shows that the two clusters are located in different quadrants, consistently with the separation observed by means of cluster analysis (Figure 6).

**Figure 7:**
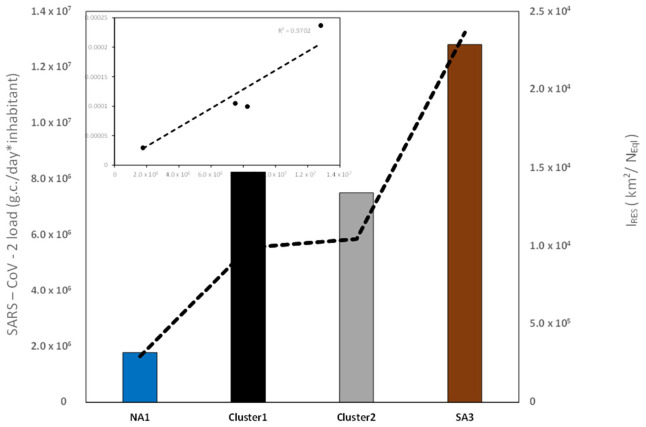
I_RES_ index and mean values of SARS-CoV-2 load calculated for each cluster. In the box the scatterplot (R^2^ = 0.9702).

To interpret our results, through external variables we introduce the *I_RES_* index, defined for each samplig site (and consequently also for each cluster) as the ratio between residential area extension and number of equivalent inhabitants, and we compare it with the mean values of SARS-CoV-2 load calculated for each cluster (centroids). As we can see from Figure 7, multivariate analysis highlights two extreme cases, namely where the SARS-CoV-2 load is higher, we also have a high value of the I_RES index, and vice versa. The scatterplot (box in Figure 7) shows an high level of positive correlation.

The I_RES_ index helps to explain how the number of equivalent inhabitants and the extension of residential areas can change the characteristics, especially in SARS-CoV-2 load values of the wastewaters that reaches the WWTP.

### 3.2. Temporal Analysis

We characterized the inter-annual variations observed between 2022, 2023, and 2024 analysing data collected in the three sites of Naples city (NA1, NA2, NA3). The variation in sampling sites over time was related to the evolution of the surveillance programme and resource optimisation during the transition from the pandemic to the post-pandemic phase. In Table 4 the descriptive statistics are summarized.

Across all three sites, SARS-CoV-2 load decreased from 2022 to 2023; however, statistically significant differences were observed only at site NA3 (*p* = 0.023). In 2024, SARS-CoV-2 loads were significantly lower than in 2022 across all sites. Residual diagnostics showed no evident patterns when plotted against fitted values or day of the year. Autocorrelation was largely removed by the CAR(1) structure, and quantile–quantile plots indicated approximate normality with some deviation in the tails. Residual plots for NA1 site are shown in Supplementary Figure S1 as a representative example; similar diagnostic patterns were observed for the other sites.

## 4. Discussion

SARS-CoV-2 and its variants have been monitored in Italy in various situations, for example in the studies of La Rosa et al. [16,17] and Veneri et al. [18] and, in recent years, attention in Italy has also been focused on other viruses, such as influenza A and B viruses [19] and adenoviruses [20].

In our study, SARS-CoV-2 showed broadly similar temporal trend in all the nine monitored sites, which means that the virus has circulated in the population in the same way, as already concluded in a previous study [9]. The only exception is SA3 WWTP, which showed higher SARS-CoV-2 load values from June 2022 onwards.

Cluster analysis showed that the WWTPs can be grouped in two clusters (Cluster 1 and Cluster 2) and in two isolated elements, SA3 and NA1 WWTP.

In particular, SA3 WWTP showed the highest average SARS-CoV-2 load values, NA1 WWTP the lowest, and the two clusters Cl1 and Cl2 occupy an intermediate position between these two WWTPs. In addition, multivariate analysis showed that a high viral load is related with a high *I_RES_* index, and vice versa.

This result could explain the different behaviour of the SARS-CoV-2 load in WWTPs. Probably, a WWTP with a high *I_RES_* index is better able to monitor variations in viral load. A large WWTP, on the other hand, could attenuate signal variability due to dilution and mixing effects.

Efforts to identify the best sites for environmental surveillance of SARS-CoV-2 have been undertaken in several other studies. For example, in the paper by Guerrero-Latorre et al., the authors describe the design and implementation of the Catalan national SARS-CoV-2 surveillance project. The study also describes the criteria for selecting the WWTPs to be monitored, considering which ones reach the largest number of inhabitants and which ones allow for the monitoring of the largest area. Ultimately, WWTPs with a number of equivalent inhabitants higher than 150,000 were selected [21]. The study by Calle et al., on the other hand, proposes algorithms for identifying sites within the sewage network to be monitored for the detection of the virus in wastewater, stating that sites should be selected that provide maximum coverage of the population and avoid overlap between them. In the case of the city of Girona (Spain), selecting 5, 6, or 7 sites makes it possible to obtain 85% coverage of population [22]. The study by Rusiñol et al. monitored the presence of SARS-CoV-2 in WWTPs of various sizes in Catalonia, observing that small WWTPs (with a number of equivalent inhabitants less than 24,000) detected lower viral load levels than large WWTPs, despite the incidence of infection being similar. The study’s authors conclude that small plants are not useful for monitoring SARS-CoV-2, as a high number of infections is required for the virus to be detected in wastewater [23].

In addition, other studies support our results. For example, in the study of Holm et al., the authors compared the cumulative concentration of SARS-CoV-2 in wastewater with population size, observing that for small populations, the cumulative concentration increased more rapidly than for large populations. Therefore, the effect of population size on the concentration of SARS-CoV-2 in wastewater is more pronounced for small populations than for large ones [24]. In the study of Pasha et al., the authors monitored the concentration of SARS-CoV-2 in two decentralised wastewater systems (serving 66 and 71 households), a large sewershed serving 1000 households, and a hospital. One of the two decentralised septic systems showed the highest detection frequency and average concentration of SARS-CoV-2, demonstrating that wastewater samples from decentralised systems can be used for continuous monitoring of the virus [25]. In addition, the study by Sartirano et al. monitored the concentration of the virus in the wastewater of a small WWTP serving 13,935 inhabitants, noting a correlation between viral concentration in wastewater and the number of infections [26]. In the study by Schmiege et al., the authors investigate the presence of SARS-CoV-2 variants in sub-sewersheds (on a small scale). In their study, they observe, among other things, that the Omicron variant appears in a sub-sewershed even before it appears in the main WWTP, highlighting the effectiveness of small-scale monitoring in tracking the presence of viral variants detailed and promptly [27].

Regarding the annual averages of SARS-CoV-2 loads in all the three WWTPs in the Naples area, from 2022 to 2024 we noted a decrease in this value, probably due to the transition from a pandemic to an endemic period during this time, with a reduction in the number of infections and, consequently, in viral shedding in wastewater. Furthermore, this reduction could be caused by the circulation of variants with different characteristics. Also in this case, the SARS-CoV-2 load in wastewater reflected the epidemiological situation, as previously observed in the study of Lombardi et al. [9].

NA3 WWTP showed a statistically significant difference in SARS-CoV-2 load from 2022 to 2023. The earliest reduction in SARS-CoV-2 load could be due to the fact that it is the smallest of the three WWTPs and, therefore, can better detect variations in viral load in wastewater over time. Overall, both spatial and temporal analyses suggest that a small WWTP better monitors variations in SARS-CoV-2 levels in wastewater.

These results allow to answer the study’s initial question, as follows: to assess whether certain WWTPs can be selected as a model for future SARS-CoV-2 surveillance, thereby optimising the number of samples to be collected. The authors suggest that WWTPs with a high CV% can provide a better indication of increases and decreases in SARS-CoV-2 loads in wastewater and, consequently, in the population. Thus, in order to optimize the monitoring network, our method suggests limiting samplings to the following three sites: SA3 (the one that showed the highest values) and then two belonging respectively to the two clusters. The first can act as a sentinel site, as it always measures the highest values and has a high CV%; observing changes at this site can indicate an increase in infections, and a biweekly sampling can be suggested. For the others, two sites can be chosen for monitoring; one selection criterion can be based on the higher CV%, so we can select CE1 for Cluster1 and NA4 for Cluster 2. The sampling sites with higher CV% are those most sensitive to variations in external conditions.

Our study has some limitations. Firstly, we cannot definitively assert that the three WWTPs truly represent the entire population. Among the future aims, there is a need (for SARS-CoV-2 as for other possible parameters) to validate the representativeness of the trends from the selected WWTPs with respect to the signals from the overall population. At the same time, this approach can also be seen as a strength in terms of exploration, as a basis for future validation studies. Finally, sampling frequency varied over time, with more frequent observations in earlier phases of the study. Although this may introduce temporal imbalance, the use of models adjusting for seasonality and temporal correlation partially mitigates this issue. The consistency of findings across sites suggests that the main conclusions are robust, although residual bias cannot be excluded.

## 5. Conclusions

Our study, based on the spatial and temporal analysis of SARS-CoV-2 at nine sites in the Campania region, allowed us to suggest, among the nine sites, three sites to focus on for future studies for SARS-CoV-2 monitoring. One of the sites, SA3, with the highest viral load values, could act as a sentinel and indicate an increase in infections through changes in its viral loads. The other two, CE1 and NA4, have a high CV% so they could be used to monitor changes in infections in the population. Multivariate analysis allows to quantify the differences observed at the various sampling sites. The site where the highest values are measured and the one where the lowest values are measured differ in a statistically significant way and can indeed be proposed as sites to monitor closely. The *I_RES_* index, based on variables different from those used by the classification algorithm, provides a possible interpretation of the result. In particular, in this case, it is important not only how many inhabitants are associated with a sewage collector but also over how much area they are distributed. However, it is important to highlight that this surveillance is based on a single regional dataset and is not supported by external validation. Further validation in other settings could be useful to confirm our results. This model could allow in the future to optimise the number of samples and to focus only on certain WWTPs with specific characteristics for SARS-CoV-2 monitoring and other pathogenic microorganisms, to promptly identify outbreaks and prevent future epidemic waves.

## Figures and Tables

**Table 1:** Urban classes extracted from CLC 2018, distinguishing between residential and commercial/industrial areas.

	Residential Areas	Commercial/ Industrial Areas
111: Continuous urban fabric	X	
112: Discontinuous urban fabric	X	
121: Industrial or commercial units		X
122: Road and rail networks and associated land		
123: Port areas		X
124: Airports		X
131: Mineral extraction sites		
132: Dump sites		
133: Construction sites		
141: Green urban areas		
142: Sport and leisure facilities		

**Table 2:** Sampling program and characteristics of sampling site. Legend: ID = sampling site label; WWTP_ID = WWTP identification; N = number of samples; Start date = date of sampling start; End date = date of sampling end; N_eqI_ = number of equivalent inhabitants; A_RES_ = extension of residential area; A_NO-RES_ = extension of commercial or industrial areas.

ID	WWTP_ID	Province	N	Start Date	End Date	N_eqI_	A_RES_ (km^2^)	A_NO_-_RES_ (km^2^)
NA1	Napoli Est	Naples	85	21 October 2021	07 May 2025	1,750,000	51.57	12.47
NA2	Napoli Ovest—Main entrance	Naples	85	21 October 2021	07 May 2025	950,000	55.89	9.63
NA3	Napoli Ovest—North sewer	Naples	84	21 October 2021	07 May 2025	250,000	47.26	3.23
NA4	Area Nolana	Naples	62	21 October 2021	14 March 2023	400,000	49.68	6.09
CE1	Area Casertana	Caserta	64	21 October 2021	14 March 2023	370,769	51.78	13.14
CE2	Villa Literno	Caserta	58	02 February 2022	14 March 2023	631,714	62.97	7.66
SA1	Salerno	Salerno	56	21 December 2021	14 March 2023	700,000	23.39	5.86
SA2	Nocera Sup.	Salerno	64	21 October 2021	14 March 2023	300,000	12.62	2.67
SA3	Eboli	Salerno	47	17 February 2022	14 March 2023	30,000	7.10	1.96

**Table 3:** PCA results for the first three principal components. The percentage of explained variance (*p*%) and the percentage contribution of each sapling sites are shown.

	PC1	PC2	PC3
*p%*	67.8	9.8	6.6
NA1	10.5	0.3	31.6
NA2	13.1	6.2	1.6
NA3	11.0	7.2	26.4
NA4	11.2	11.6	5.4
CE1	9.7	13.4	13.9
SA2	12.8	8.2	5.9
SA1	12.2	0.2	12.6
SA3	6.3	51.4	0.0
CE2	13.1	1.3	2.6

**Table 4:** SARS-CoV-2 loads across the three monitoring sites by year. Values are expressed as median and interquartile range (IQR). ND indicates non-detectable samples. *p*-values refer to pairwise comparisons between years obtained from generalized least squares models accounting for temporal correlation and adjusting for seasonality (day of the year).

Site	Year	*n*	Median [IQR]	ND (%)	*p* Value
NA1	2022	48	1.05 × 10^6^ [2.82 × 10^5^; 2.22 × 10^6^]	1 (2.1%)	-
	2023	11	5.54 × 10^5^ [1.42 × 10^5^; 7.91 × 10^5^]	1 (9.1%)	vs. 2022: 0.097;
	2024	11	3.54 × 10^5^ [ND; 1.11 × 10^6^]	4 (36.4%)	vs. 2022: 0.001; vs. 2023: 0.183
NA2	2022	48	6.28 × 10^6^ [1.90 × 10^6^; 1.47 × 10^7^]	0 (0%)	-
	2023	11	3.13 × 10^6^ [6.93 × 10^5^; 6.71 × 10^6^]	0 (0%)	vs. 2022: 0.082
	2024	11	9.52 × 10^4^ [ND; 3.80 × 10^6^]	2 (18.2%)	vs. 2022: <0.001; vs. 2023: 0.029
NA3	2022	48	5.42 × 10^6^ [2.44 × 10^6^; 1.07 × 10^7^]	0 (0%)	-
	2023	10	2.81 × 10^6^ [4.53 × 10^5^; 4.33 × 10^6^]	0 (0%)	vs. 2022: 0.023;
	2024	11	1.14 × 10^6^ [ND; 3.45 × 10^6^]	3 (27.3%)	vs. 2022: <0.001; vs. 2023: 0.078

## Data Availability

The original contributions presented in this study are included in the article. Further inquiries can be directed to the corresponding author.

## Supplementary Materials

The following supporting information can be downloaded at: https://www.mdpi.com/article/10.3390/microorganisms14051063/s1, Figure S1: Diagnostic plots for the generalized least squares model fitted for site NA1. Panel A shows normalized residuals plotted against the day of the year to assess residual seasonal patterns. Panel B shows the normal Q–Q plot of normalized residuals. Panel C shows the autocorrelation function (ACF) of normalized residuals used to evaluate the adequacy of the CAR(1) correlation structure. NA1 was selected as a representative example; similar diagnostic patterns were observed for the other sites.
