# Supplementary material for: Multidimensional Analysis of SARS-CoV-2 RNA in Nine Sites Located in Campania Region, Italy
Source: Microorganisms. 2026 May 8;14(5):1063. doi: 10.3390/microorganisms14051063 (PMC13209735; doi:10.3390/microorganisms14051063)

Model diagnostic plots NA1

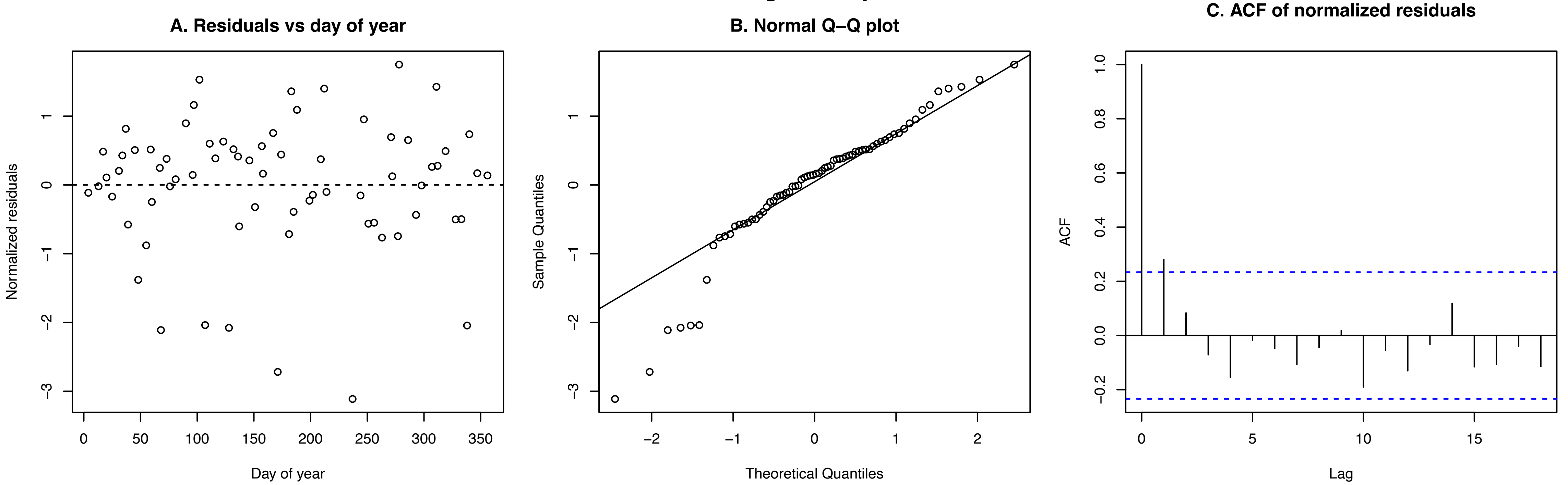

Supplement: Supplementary file 1 [file microorganisms-14-01063-s001.zip › microorganisms-4291461-supplementary.pdf]
